# Supplementary material for: Sodium Solid Electrolytes: NaxAlOy Bilayer-System Based on Macroporous Bulk Material and Dense Thin-Film
Source: Materials (Basel). 2021 Feb 10;14(4):854. doi: 10.3390/ma14040854 (PMC7916714; doi:10.3390/ma14040854)
Supplement: Supplementary file 1 [file materials-14-00854-s001.zip › materials-1077999-supplementary.pdf]

materials

MDPI

Supplementary Materials

Sodium Solid Electrolytes: Na_xAlO_y Bilayer-System Based on Macroporous Bulk Material and Dense Thin-Film

Antonia Hoppe [1], Cornelius Dirksen [2], Karl Skadell [2], Michael Stelter [2], Matthias Schulz [2], Simon Carstens [1], Dirk Enke [1] and Sharon Koppka [1,*]

[1] Institute of Chemical Technology, Universität Leipzig, Leipzig, Germany; antonia.hoppe@uni-leipzig.de (A.H.); simon.carstens@uni-leipzig.de (S.C.); dirk.enke@uni-leipzig.de (D.E.)
[2] Fraunhofer Institute for Ceramic Technologies and Systems IKTS, Michael-Faraday-Str. 1, 07629 Hermsdorf, Germany; cornelius.dirksen@ikts.fraunhofer.de (C.D.); karl.skadell@ikts.fraunhofer.de (K.S.); michael.stelter@ikts.fraunhofer.de (M.S.); matthias.schulz@ikts.fraunhofer.de (M.S.)
* Correspondence: sharon.koppka@uni-leipzig.de

Table S1. Spin-coating regime to coat calcined discs.

Step-Nr.	Function	Rotation Speed/rpm	Duration/s
1	check position	200	10
2	deposition of slurry	800	5
3	Spinning	2000	30
4	Drying	800	20

Table S2. The modal pore size (d_{mod}), pore volume (V_P) and porosity (P) were determined by mercury intrusion measurement of calcined xerogels with different initial PEO amount. The detected pore structure above 100 nm is generated by the interparticle space and was hence omitted for the calculation of pore volume and porosity. The uncertainty of the measurement is ±5% of the modal pore size, ±0.05 $cm^3 \cdot g^{-1}$ for pore volume and ±2% for porosity.

m_{PEO}/g	d_{mod}/nm	V_P/$cm^3 \cdot g^{-1}$	P/%
0.00	22	0.41	59
0.01	21	0.43	61
0.10	21	0.41	59

Table S3. The modal pore size (d_{mod}), pore volume (V_P) and porosity (P) were determined by mercury intrusion measurement of the untreated disc with different initial PEO amounts (m_{PEO}). See table S2 for uncertainty.

m_{PEO}/g	d_{mod}/nm	V_P/$cm^3 \cdot g^{-1}$	P/%
0.00	18	0.33	54
0.01	16	0.31	52
0.10	17	0.37	57

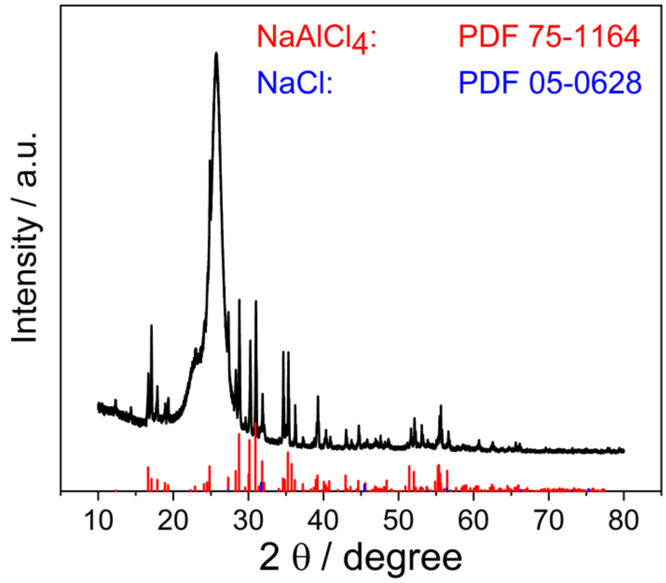

Figure S1. XRD pattern of $NaAlCl_4$ (black). The red and the blue reflexes represent the literature data for $NaAlCl_4$ (ICDD-database PDF 75-1164) and NaCl (ICDD-database PDF 05-0628). The dominant reflex at 25° is caused by an X-ray foil that protected the sample from moisture.

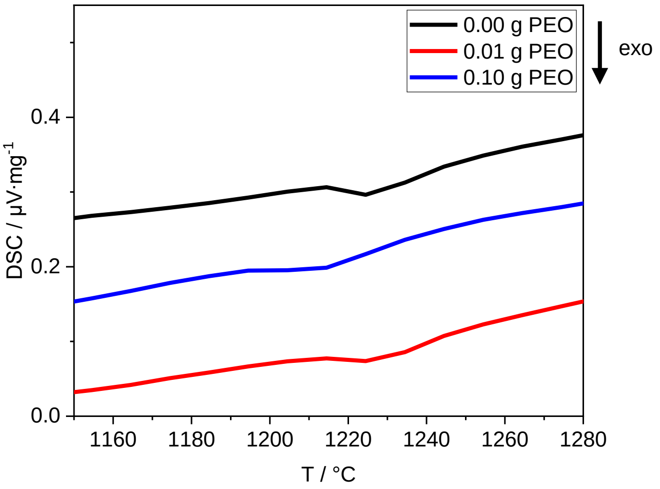

Figure S2. Magnification of the DSC curves of the xerogels from Figure 4b, recorded with a heating rate of 10 $K \cdot min^{-1}$. The sample without PEO is labeled black, the one containing 0.01 g PEO is labeled red. The sample labeled blue initially contained 0.10 g PEO and shows a shift for the exothermic peak around 1200 °C towards a slightly lower temperature.

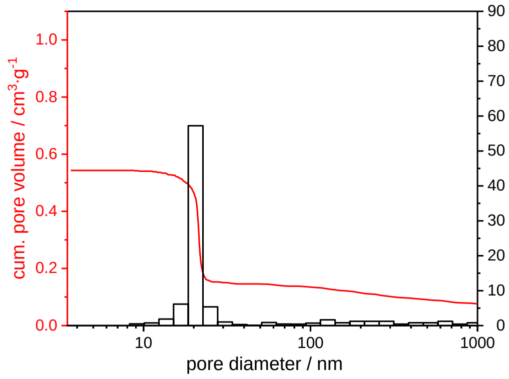

(**a**)

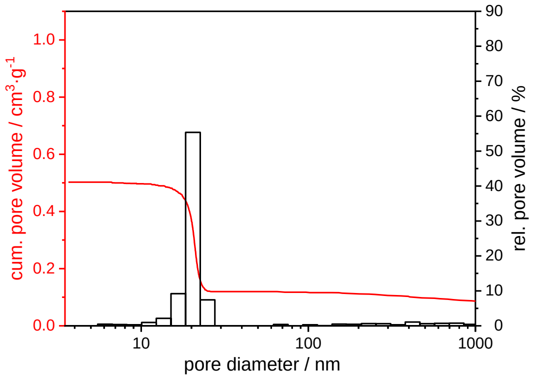

(**b**)

Figure S3. Cumulative (red) and relative pore volume (black) from the mercury intrusion of xerogels with (**a**) 0.00 g and (**b**) 0.10 g initial PEO.

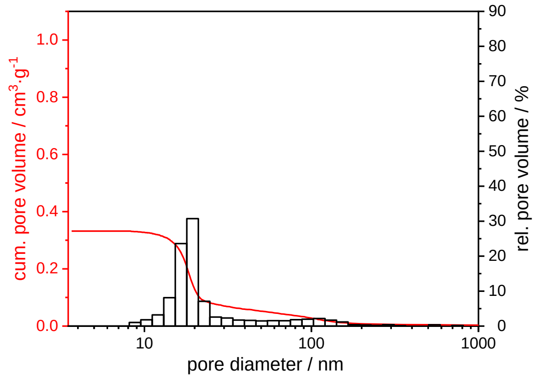

(**a**)

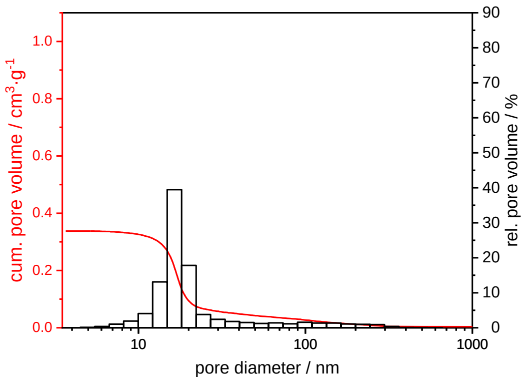

(**b**)

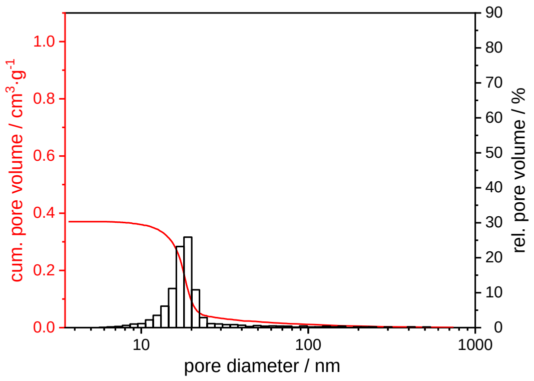

(**c**)

Figure S4. Cumulative (red) and relative pore volume (black) from the mercury intrusion of the untreated disc with (**a**) 0.00 g (**b**) 0.01 g and (**c**) 0.10 g initial PEO.

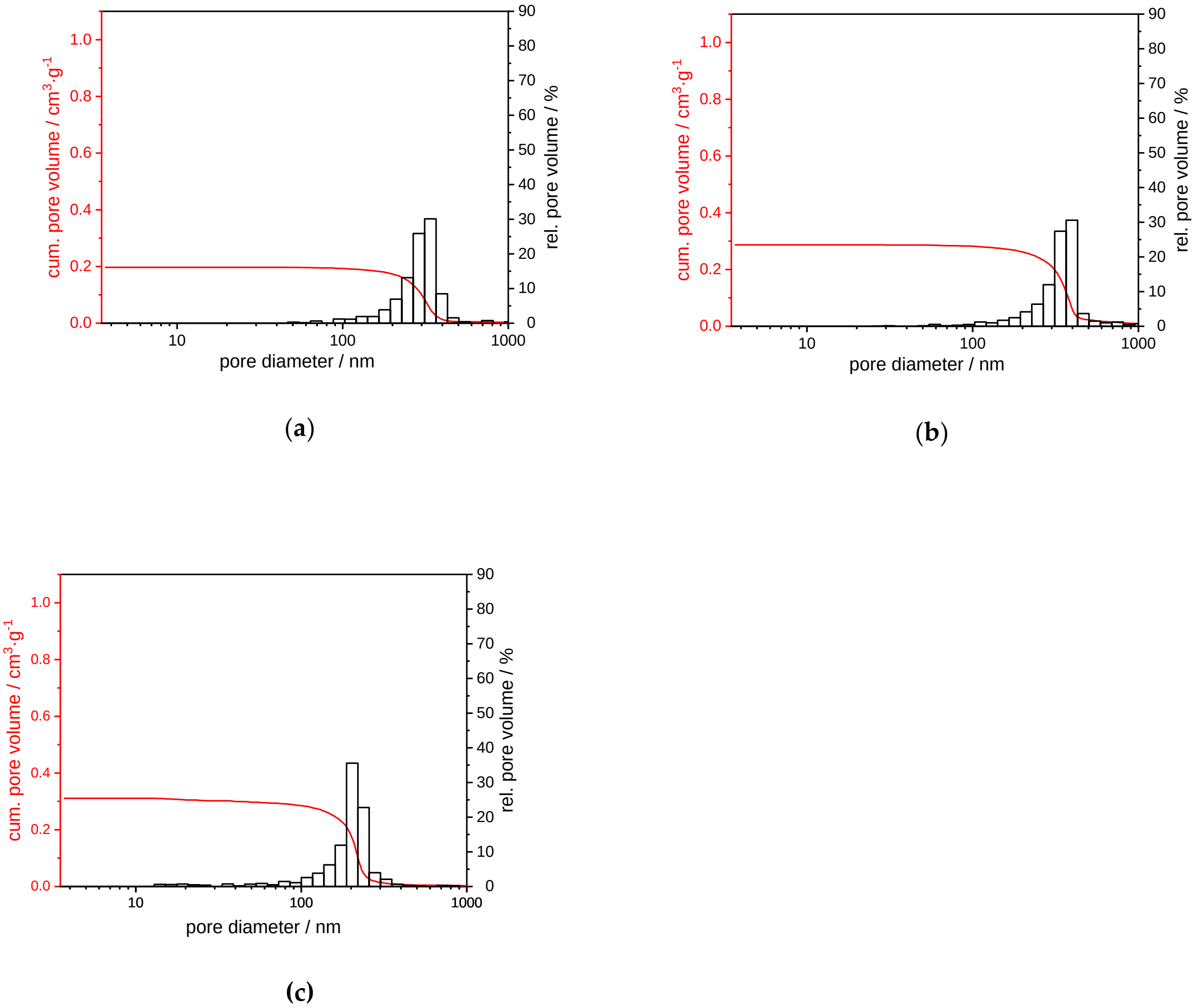

Figure S5. Cumulative (red) and relative pore volume (black) from the mercury intrusion of the disc heat treated at 1400 °C for 2 h with (**a**) 0.00 g (**b**) 0.01 g and (**c**) 0.10 g initial PEO.

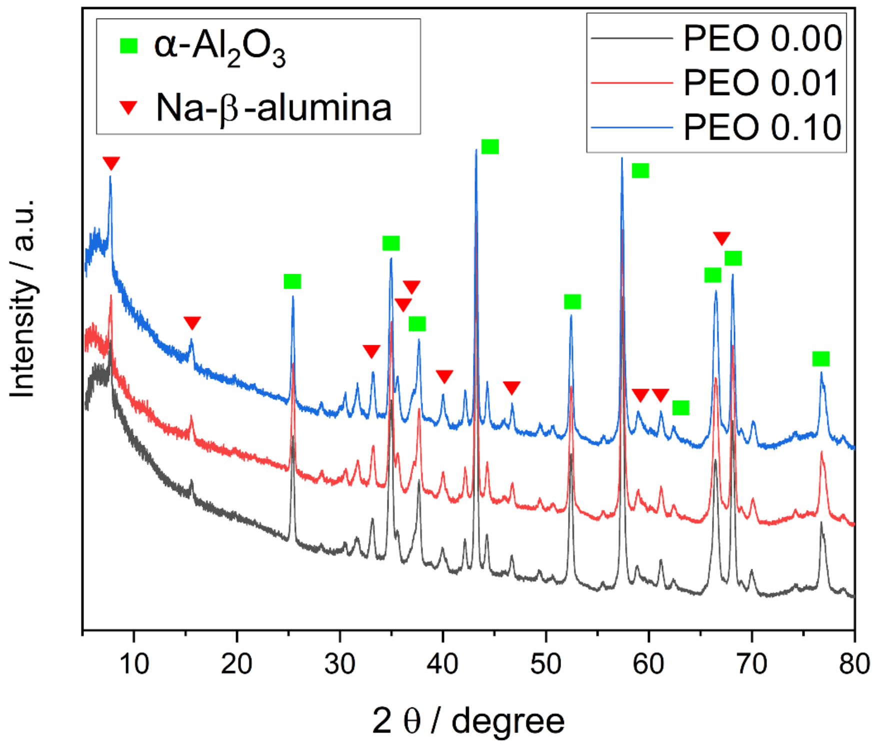

Figure S6. XRD patterns of BEs synthesized with different initial PEO amounts of 0.00 g PEO (black), 0.01 g (red) and 0.01 g (blue). The main reflexes α-Al_2O_3 and Na-β-alumina are labeled with green squares and red triangles, respectively.
